# 2-[(1*H*-Imidazol-1-yl)meth­yl]-1-[4-(trifluoro­meth­yl)phen­yl]-1*H*-indole

**DOI:** 10.1107/S1600536812010471

**Published:** 2012-03-17

**Authors:** Rui Wang, Hong-fan Shi, Lin Du, Jing-feng Zhao, Jian-ping Liu

**Affiliations:** a Key Laboratory of Medicinal Chemistry for Natural Resource, Ministry of Education, School of Chemical Science and Technology, Yunnan University, Kunming 650091, People’s Republic of China

## Abstract

In the title compound, C_19_H_14_F_3_N_3_, the dihedral angles between the mean planes of the indole ring and the 4-CF_3_-phenyl and imidazole rings are 54.95 (4) and 61.36 (7)°, respectively.

## Related literature

For background to indole derivatives and their biological activity, see: Muftuoglua & Mustatab (2010 ▶); Jiao *et al.* (2010 ▶). For related structures, see: Borgne *et al.* (1999 ▶); Lézé *et al.* (2006 ▶); Marchand *et al.* (2003 ▶).
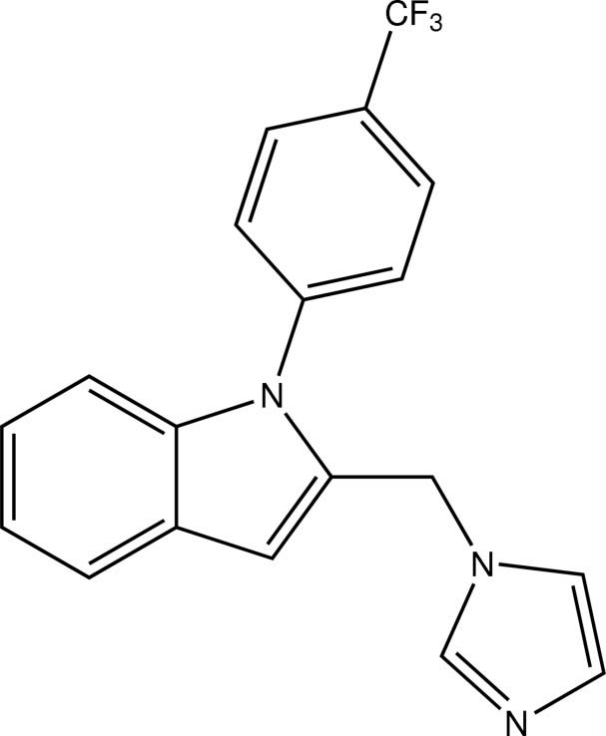


## Experimental

### 

#### Crystal data

C_19_H_14_F_3_N_3_
*M* *_r_* = 341.33
Orthorhombic, 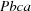

*a* = 10.3732 (17) Å
*b* = 7.9960 (13) Å
*c* = 37.665 (6) Å
*V* = 3124.0 (9) Å^3^
*Z* = 8
Mo *K*α radiation
μ = 0.11 mm^−1^
*T* = 100 K
0.53 × 0.26 × 0.05 mm

#### Data collection

Bruker APEXII CCD diffractometer
Absorption correction: multi-scan (*SADABS*; Sheldrick, 2004 ▶) *T*_min_ = 0.943, *T*_max_ = 0.994
30000 measured reflections
4505 independent reflections
3217 reflections with *I* > 2σ(*I*)
*R*_int_ = 0.093

#### Refinement

*R*[*F* ^2^ > 2σ(*F* ^2^)] = 0.079
*wR*(*F* ^2^) = 0.214
*S* = 0.99
4505 reflections
226 parameters
H-atom parameters constrained
Δρ_max_ = 0.51 e Å^−3^
Δρ_min_ = −0.38 e Å^−3^

### 

Data collection: *APEX2* (Bruker, 1998 ▶); cell refinement: *SAINT* (Bruker, 1998 ▶); data reduction: *SAINT*; program(s) used to solve structure: *SHELXS97* (Sheldrick, 2008 ▶); program(s) used to refine structure: *SHELXL97* (Sheldrick, 2008 ▶); molecular graphics: *SHELXTL* (Sheldrick, 2008 ▶); software used to prepare material for publication: *SHELXTL*.

## Supplementary Material

Crystal structure: contains datablock(s) I, global. DOI: 10.1107/S1600536812010471/jj2125sup1.cif

Structure factors: contains datablock(s) I. DOI: 10.1107/S1600536812010471/jj2125Isup2.hkl

Supplementary material file. DOI: 10.1107/S1600536812010471/jj2125Isup3.cml

Additional supplementary materials:  crystallographic information; 3D view; checkCIF report

## Figures and Tables

**Fig. 1.**
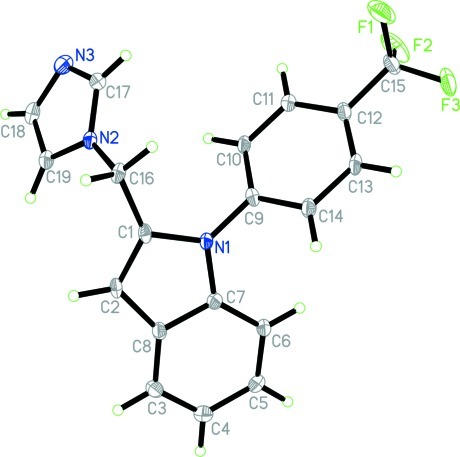
The molecular structure of (I) showing the atomic numbering and 50% probability displacement ellipsoids.

**Fig. 2.**
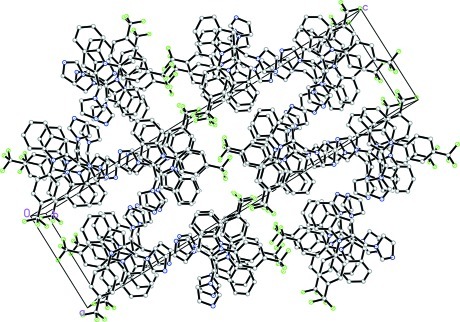
The crystal packing of (I) viewed along the b axis. H atoms have been removed for clarity.

## supplementary crystallographic information

### Comment

Iodole derivatives are an important class of heterocycles in medicinal chemistry (Borgne *et al.*, 1999; Marchand *et al.*, 2003; Lézé *et al.*, 2006; Jiao *et al.*, 2010; Muftuoglua & Mustatab, 2010). In continuation of our studies on N-aromatization in the indole ring, we present here the the crystal structure of the title compound, C_19_H_14_F_3_N_3_, (I). With one molecule in the asymmetric unit, the dihedral angles between the mean planes of the indole ring and the 4-CF_3_-phenyl and imidazole rings are 54.95 (4)° and 61.36 (7)°, respectively (Fig. 1). Bond lengths and angles are in normal ranges.

### Experimental

The title compound, C_19_H_14_F_3_N_3_, was prepared in one step. (1-(4-(trifluoromethyl)phenyl)-1*H*-indol-2-yl)methanol (50 mg, 0.17 mmol) and *N*,*N*'-carbonyldiimidazole (83 mg, 3 equiv) in dry CH_3_CN was stirred for 48 h at room temperature. The solvent was evaporated and the residue purified on silica gel to give the title compound,(I). Yield: 53% (33 mg). Recrystallization from absolute ethyl acetate gave colorless and clear single crystals for X-ray diffraction measurement.

### Refinement

H-atoms were placed in calculated positions [C—H = 0.99 Å for aliphatic H, 0.95 Å for aromatic H, *U*_iso_(H)= 1.2*U*_eq_(C)] and were included in the refinement in the riding model approximation.

### Crystal data

**Table:**

C_19_H_14_F_3_N_3_	*F*(000) = 1408
*M**_r_* = 341.33	*D*_x_ = 1.451 Mg m^−^^3^
Orthorhombic, *P**b**c**a*	Mo *K*α radiation, λ = 0.71073 Å
Hall symbol: -P 2ac 2ab	Cell parameters from 5075 reflections
*a* = 10.3732 (17) Å	θ = 2.2–30.1°
*b* = 7.9960 (13) Å	µ = 0.11 mm^−^^1^
*c* = 37.665 (6) Å	*T* = 100 K
*V* = 3124.0 (9) Å^3^	Laminiplantation, colourless
*Z* = 8	0.53 × 0.26 × 0.05 mm

### Data collection

**Table:**

Bruker APEXII CCD diffractometer	4505 independent reflections
Radiation source: fine-focus sealed tube	3217 reflections with *I* > 2σ(*I*)
Graphite monochromator	*R*_int_ = 0.093
Detector resolution: 0.71 pixels mm^-1^	θ_max_ = 30.2°, θ_min_ = 1.1°
φ and ω scans	*h* = −14→14
Absorption correction: multi-scan (*SADABS*; Sheldrick, 2004)	*k* = −11→11
*T*_min_ = 0.943, *T*_max_ = 0.994	*l* = −52→52
30000 measured reflections	

### Refinement

**Table:**

Refinement on *F*^2^	Primary atom site location: structure-invariant direct methods
Least-squares matrix: full	Secondary atom site location: difference Fourier map
*R*[*F*^2^ > 2σ(*F*^2^)] = 0.079	Hydrogen site location: inferred from neighbouring sites
*wR*(*F*^2^) = 0.214	H-atom parameters constrained
*S* = 0.99	*w* = 1/[σ^2^(*F*_o_^2^) + (0.0877*P*)^2^ + 8.8995*P*] where *P* = (*F*_o_^2^ + 2*F*_c_^2^)/3
4505 reflections	(Δ/σ)_max_ = 0.001
226 parameters	Δρ_max_ = 0.51 e Å^−^^3^
0 restraints	Δρ_min_ = −0.38 e Å^−^^3^

### Special details

**Table:**

Geometry. All e.s.d.'s (except the e.s.d. in the dihedral angle between two l.s. planes) are estimated using the full covariance matrix. The cell e.s.d.'s are taken into account individually in the estimation of e.s.d.'s in distances, angles and torsion angles; correlations between e.s.d.'s in cell parameters are only used when they are defined by crystal symmetry. An approximate (isotropic) treatment of cell e.s.d.'s is used for estimating e.s.d.'s involving l.s. planes.
Refinement. Refinement of *F*^2^ against ALL reflections. The weighted *R*-factor *wR* and goodness of fit *S* are based on *F*^2^, conventional *R*-factors *R* are based on *F*, with *F* set to zero for negative *F*^2^. The threshold expression of *F*^2^ > σ(*F*^2^) is used only for calculating *R*-factors(gt) *etc*. and is not relevant to the choice of reflections for refinement. *R*-factors based on *F*^2^ are statistically about twice as large as those based on *F*, and *R*- factors based on ALL data will be even larger.

### Fractional atomic coordinates and isotropic or equivalent isotropic displacement parameters (Å^2^)

**Table:**

	*x*	*y*	*z*	*U*_iso_*/*U*_eq_	
F1	−0.1652 (2)	0.1525 (3)	0.97255 (6)	0.0444 (6)	
F2	−0.1194 (2)	0.4118 (3)	0.97874 (6)	0.0422 (6)	
F3	−0.0238 (2)	0.2278 (3)	1.01033 (5)	0.0430 (6)	
N1	0.3126 (2)	0.1845 (3)	0.86957 (5)	0.0130 (4)	
N2	0.0946 (2)	0.1498 (3)	0.80082 (6)	0.0141 (4)	
N3	−0.0810 (2)	0.2676 (3)	0.77776 (7)	0.0229 (5)	
C1	0.2961 (3)	0.1098 (3)	0.83609 (6)	0.0133 (5)	
C2	0.4120 (3)	0.1060 (3)	0.81894 (6)	0.0143 (5)	
H2	0.4275	0.0596	0.7961	0.017*	
C3	0.6355 (3)	0.2300 (4)	0.83646 (7)	0.0192 (6)	
H3	0.6802	0.1996	0.8154	0.023*	
C4	0.6967 (3)	0.3203 (4)	0.86292 (8)	0.0228 (6)	
H4	0.7839	0.3535	0.8598	0.027*	
C5	0.6313 (3)	0.3638 (4)	0.89460 (7)	0.0215 (6)	
H5	0.6758	0.4248	0.9124	0.026*	
C6	0.5041 (3)	0.3194 (4)	0.90018 (7)	0.0167 (5)	
H6	0.4604	0.3484	0.9215	0.020*	
C7	0.4424 (2)	0.2299 (3)	0.87310 (7)	0.0147 (5)	
C8	0.5063 (3)	0.1844 (3)	0.84135 (7)	0.0151 (5)	
C9	0.2173 (3)	0.2037 (3)	0.89657 (7)	0.0140 (5)	
C10	0.1007 (2)	0.2828 (3)	0.88912 (7)	0.0144 (5)	
H10	0.0841	0.3242	0.8659	0.017*	
C11	0.0090 (3)	0.3010 (4)	0.91571 (7)	0.0168 (5)	
H11	−0.0712	0.3530	0.9106	0.020*	
C12	0.0349 (3)	0.2429 (3)	0.94987 (7)	0.0160 (5)	
C13	0.1510 (3)	0.1651 (3)	0.95761 (7)	0.0170 (5)	
H13	0.1679	0.1257	0.9809	0.020*	
C14	0.2428 (3)	0.1450 (3)	0.93089 (6)	0.0164 (5)	
H14	0.3225	0.0916	0.9360	0.020*	
C15	−0.0667 (3)	0.2588 (4)	0.97782 (7)	0.0205 (6)	
C16	0.1706 (3)	0.0383 (3)	0.82356 (7)	0.0152 (5)	
H16A	0.1884	−0.0661	0.8103	0.018*	
H16B	0.1181	0.0083	0.8446	0.018*	
C17	−0.0343 (3)	0.1775 (4)	0.80402 (7)	0.0189 (5)	
H17	−0.0849	0.1364	0.8231	0.023*	
C18	0.0239 (3)	0.2973 (4)	0.75601 (7)	0.0202 (6)	
H18	0.0207	0.3596	0.7346	0.024*	
C19	0.1328 (3)	0.2251 (3)	0.76948 (7)	0.0166 (5)	
H19	0.2169	0.2264	0.7595	0.020*	

### Atomic displacement parameters (Å^2^)

**Table:**

	*U*^11^	*U*^22^	*U*^33^	*U*^12^	*U*^13^	*U*^23^
F1	0.0345 (11)	0.0602 (15)	0.0383 (12)	−0.0245 (11)	0.0181 (9)	−0.0139 (11)
F2	0.0478 (13)	0.0346 (11)	0.0444 (12)	0.0137 (10)	0.0285 (10)	0.0034 (10)
F3	0.0367 (11)	0.0788 (17)	0.0134 (9)	0.0105 (11)	0.0078 (8)	0.0042 (10)
N1	0.0148 (10)	0.0162 (10)	0.0081 (9)	0.0008 (8)	−0.0002 (8)	0.0005 (8)
N2	0.0169 (11)	0.0151 (10)	0.0105 (10)	−0.0008 (8)	−0.0006 (8)	−0.0022 (8)
N3	0.0195 (12)	0.0278 (13)	0.0215 (12)	0.0005 (10)	−0.0042 (10)	−0.0042 (10)
C1	0.0195 (12)	0.0123 (11)	0.0082 (10)	0.0026 (9)	0.0004 (9)	0.0018 (9)
C2	0.0207 (13)	0.0143 (11)	0.0079 (10)	0.0023 (10)	0.0011 (9)	−0.0015 (9)
C3	0.0161 (13)	0.0258 (14)	0.0157 (12)	0.0045 (11)	0.0025 (10)	0.0014 (11)
C4	0.0149 (12)	0.0335 (16)	0.0202 (13)	−0.0007 (11)	0.0004 (10)	−0.0006 (12)
C5	0.0167 (13)	0.0314 (15)	0.0165 (13)	−0.0001 (11)	−0.0042 (10)	−0.0047 (11)
C6	0.0173 (12)	0.0213 (13)	0.0114 (11)	0.0022 (10)	0.0001 (9)	−0.0021 (10)
C7	0.0145 (11)	0.0171 (12)	0.0123 (11)	0.0021 (9)	−0.0007 (9)	0.0008 (9)
C8	0.0172 (12)	0.0177 (12)	0.0104 (11)	0.0040 (10)	−0.0001 (9)	−0.0002 (9)
C9	0.0186 (12)	0.0132 (11)	0.0102 (11)	−0.0002 (9)	0.0027 (9)	−0.0011 (9)
C10	0.0177 (12)	0.0170 (12)	0.0085 (11)	−0.0010 (10)	−0.0009 (9)	0.0014 (9)
C11	0.0163 (12)	0.0216 (13)	0.0125 (12)	0.0004 (10)	−0.0007 (9)	−0.0006 (10)
C12	0.0188 (12)	0.0188 (12)	0.0104 (11)	−0.0032 (10)	0.0021 (10)	−0.0014 (9)
C13	0.0236 (13)	0.0197 (13)	0.0076 (11)	−0.0016 (10)	−0.0014 (10)	0.0015 (9)
C14	0.0190 (12)	0.0196 (13)	0.0106 (11)	0.0023 (10)	−0.0005 (10)	0.0009 (10)
C15	0.0214 (13)	0.0254 (15)	0.0148 (12)	−0.0026 (11)	0.0068 (10)	−0.0013 (10)
C16	0.0209 (13)	0.0144 (12)	0.0103 (11)	−0.0026 (10)	−0.0023 (10)	0.0006 (9)
C17	0.0148 (12)	0.0262 (14)	0.0157 (12)	−0.0012 (11)	0.0008 (10)	−0.0039 (11)
C18	0.0271 (14)	0.0198 (13)	0.0136 (12)	0.0003 (11)	−0.0043 (11)	−0.0019 (10)
C19	0.0208 (13)	0.0175 (12)	0.0115 (11)	−0.0012 (10)	−0.0006 (10)	−0.0003 (10)

### Geometric parameters (Å, º)

**Table:**

F1—C15	1.344 (4)	C5—H5	0.9500
F2—C15	1.340 (4)	C6—C7	1.401 (4)
F3—C15	1.326 (3)	C6—H6	0.9500
N1—C7	1.400 (3)	C7—C8	1.415 (4)
N1—C1	1.406 (3)	C9—C10	1.393 (4)
N1—C9	1.427 (3)	C9—C14	1.400 (3)
N2—C17	1.360 (3)	C10—C11	1.389 (4)
N2—C19	1.383 (3)	C10—H10	0.9500
N2—C16	1.466 (3)	C11—C12	1.394 (4)
N3—C17	1.316 (4)	C11—H11	0.9500
N3—C18	1.383 (4)	C12—C13	1.387 (4)
C1—C2	1.365 (4)	C12—C15	1.495 (4)
C1—C16	1.498 (4)	C13—C14	1.395 (4)
C2—C8	1.436 (4)	C13—H13	0.9500
C2—H2	0.9500	C14—H14	0.9500
C3—C4	1.385 (4)	C16—H16A	0.9900
C3—C8	1.401 (4)	C16—H16B	0.9900
C3—H3	0.9500	C17—H17	0.9500
C4—C5	1.416 (4)	C18—C19	1.366 (4)
C4—H4	0.9500	C18—H18	0.9500
C5—C6	1.382 (4)	C19—H19	0.9500
			
C7—N1—C1	108.2 (2)	C11—C10—H10	120.1
C7—N1—C9	124.8 (2)	C9—C10—H10	120.1
C1—N1—C9	126.9 (2)	C10—C11—C12	119.9 (3)
C17—N2—C19	106.6 (2)	C10—C11—H11	120.0
C17—N2—C16	125.1 (2)	C12—C11—H11	120.0
C19—N2—C16	127.5 (2)	C13—C12—C11	120.7 (2)
C17—N3—C18	104.4 (2)	C13—C12—C15	120.2 (2)
C2—C1—N1	109.0 (2)	C11—C12—C15	119.0 (3)
C2—C1—C16	127.4 (2)	C12—C13—C14	119.5 (2)
N1—C1—C16	123.4 (2)	C12—C13—H13	120.2
C1—C2—C8	108.2 (2)	C14—C13—H13	120.2
C1—C2—H2	125.9	C13—C14—C9	119.9 (2)
C8—C2—H2	125.9	C13—C14—H14	120.1
C4—C3—C8	118.7 (3)	C9—C14—H14	120.1
C4—C3—H3	120.7	F3—C15—F2	106.5 (2)
C8—C3—H3	120.7	F3—C15—F1	105.9 (2)
C3—C4—C5	121.0 (3)	F2—C15—F1	105.7 (3)
C3—C4—H4	119.5	F3—C15—C12	113.4 (2)
C5—C4—H4	119.5	F2—C15—C12	112.5 (2)
C6—C5—C4	121.5 (3)	F1—C15—C12	112.2 (2)
C6—C5—H5	119.3	N2—C16—C1	114.8 (2)
C4—C5—H5	119.3	N2—C16—H16A	108.6
C5—C6—C7	117.2 (2)	C1—C16—H16A	108.6
C5—C6—H6	121.4	N2—C16—H16B	108.6
C7—C6—H6	121.4	C1—C16—H16B	108.6
N1—C7—C6	129.9 (2)	H16A—C16—H16B	107.6
N1—C7—C8	107.7 (2)	N3—C17—N2	112.6 (3)
C6—C7—C8	122.1 (2)	N3—C17—H17	123.7
C3—C8—C7	119.5 (2)	N2—C17—H17	123.7
C3—C8—C2	133.5 (2)	C19—C18—N3	111.0 (2)
C7—C8—C2	106.8 (2)	C19—C18—H18	124.5
C10—C9—C14	120.2 (2)	N3—C18—H18	124.5
C10—C9—N1	120.5 (2)	C18—C19—N2	105.3 (2)
C14—C9—N1	119.4 (2)	C18—C19—H19	127.4
C11—C10—C9	119.8 (2)	N2—C19—H19	127.4
			
C7—N1—C1—C2	0.6 (3)	C14—C9—C10—C11	−1.0 (4)
C9—N1—C1—C2	−176.1 (2)	N1—C9—C10—C11	−179.8 (2)
C7—N1—C1—C16	177.2 (2)	C9—C10—C11—C12	1.2 (4)
C9—N1—C1—C16	0.4 (4)	C10—C11—C12—C13	−0.8 (4)
N1—C1—C2—C8	−1.6 (3)	C10—C11—C12—C15	−178.1 (3)
C16—C1—C2—C8	−178.0 (2)	C11—C12—C13—C14	0.1 (4)
C8—C3—C4—C5	1.0 (5)	C15—C12—C13—C14	177.4 (3)
C3—C4—C5—C6	−0.6 (5)	C12—C13—C14—C9	0.1 (4)
C4—C5—C6—C7	−0.2 (4)	C10—C9—C14—C13	0.4 (4)
C1—N1—C7—C6	174.3 (3)	N1—C9—C14—C13	179.1 (2)
C9—N1—C7—C6	−8.9 (4)	C13—C12—C15—F3	14.2 (4)
C1—N1—C7—C8	0.6 (3)	C11—C12—C15—F3	−168.5 (3)
C9—N1—C7—C8	177.4 (2)	C13—C12—C15—F2	135.1 (3)
C5—C6—C7—N1	−172.3 (3)	C11—C12—C15—F2	−47.5 (4)
C5—C6—C7—C8	0.6 (4)	C13—C12—C15—F1	−105.8 (3)
C4—C3—C8—C7	−0.6 (4)	C11—C12—C15—F1	71.6 (4)
C4—C3—C8—C2	173.6 (3)	C17—N2—C16—C1	−134.7 (3)
N1—C7—C8—C3	174.1 (2)	C19—N2—C16—C1	56.1 (3)
C6—C7—C8—C3	−0.2 (4)	C2—C1—C16—N2	−86.5 (3)
N1—C7—C8—C2	−1.5 (3)	N1—C1—C16—N2	97.6 (3)
C6—C7—C8—C2	−175.8 (2)	C18—N3—C17—N2	1.0 (3)
C1—C2—C8—C3	−172.8 (3)	C19—N2—C17—N3	−1.5 (3)
C1—C2—C8—C7	1.9 (3)	C16—N2—C17—N3	−172.5 (2)
C7—N1—C9—C10	129.0 (3)	C17—N3—C18—C19	−0.2 (3)
C1—N1—C9—C10	−54.7 (4)	N3—C18—C19—N2	−0.7 (3)
C7—N1—C9—C14	−49.7 (4)	C17—N2—C19—C18	1.2 (3)
C1—N1—C9—C14	126.5 (3)	C16—N2—C19—C18	172.0 (2)
